# Controlled Formation of Au Nanonetworks via Discrete BTA-Oligo(Acrylic Acid)_3_ Supramolecular Templates

**DOI:** 10.3390/polym17121662

**Published:** 2025-06-15

**Authors:** Sadaf Aiman, Soonyoung Choi, Hyosun Lee, Sang-Ho Lee, Eunyong Seo

**Affiliations:** 1 Center for Specialty Chemicals, Korea Research Institute of Chemical Technology, Ulsan 44412, Republic of Korea; aiman.sadaf@sns.nust.edu.pk (S.A.); nero0407@unist.ac.kr (S.C.); 2 Department of Chemistry, School of Natural Sciences, National University of Sciences and Technology, Islamabad 44000, Pakistan; 3 Department of Chemistry and Green-Nano Materials Research Center, Kyungpook National University, 80 Daehakro, Bukgu, Daegu 41566, Republic of Korea; hyosunlee@knu.ac.kr; 4 Department of Chemical and Biochemical Engineering, Dongguk University, Seoul 04620, Republic of Korea; 5 Department of Chemical Engineering, Ulsan College, Ulsan 44610, Republic of Korea

**Keywords:** supramolecular polymer templates, BTA-oligomers, molecular dispersity, self-assembly, nanoparticle synthesis

## Abstract

Precise control over molecular dispersity and supramolecular assembly is essential for designing nanostructures with targeted properties and functionalities. In this study, we explore the impact of molecular dispersity in BTA-oligo(AA)_3_ oligomers on the formation and structural organization of Au nanomaterials in an aqueous system. Discrete and polydisperse BTA-oligo(AA)_3_ samples are systematically synthesized and characterized to evaluate their role as templates for nanostructure formation. UV-vis spectroscopy and TEM analyses reveal distinct differences in the resulting nanostructures. Specifically, discrete oligomers facilitate the formation of well-defined, interconnected Au nanonetworks with high structural uniformity, even at elevated concentrations. In contrast, polydisperse oligomers facilitated the formation of isolated Au nanoparticles with limited control over morphology and connectivity. These differences are attributed to the greater molecular uniformity and enhanced self-assembly capabilities of the discrete oligomers, which serve as effective templates for directing Au precursor organization and reduction into ordered nanostructures. This study provides mechanistic insight into how molecular dispersity affects the templating and assembly of gold nanomaterials. The findings offer a promising strategy for developing tailored nanostructures with interconnected morphologies and controlled optical and structural properties, paving the way for advanced applications.

## 1. Introduction

Precise molecular engineering of supramolecular polymers enables dispersity-dependent control over Au nanostructure formation. This study demonstrates that discrete BTA-oligo(AA)_3_ oligomers with uniform chain length serve as robust supramolecular templates, guiding the assembly of interconnected gold nanonetworks with high structural fidelity, while their polydisperse analogs yield isolated nanoparticles due to reduced templating capability.

The synthesis and development of gold nanomaterials have attracted significant attention due to their remarkable properties and broad applications in biomedicine, catalysis, optics, and electronic devices [1,2,3,4]. The functional performance of gold nanoparticles (Au NPs) depends strongly on their size, shape, and assembly, which makes precise control over these parameters essential [5,6,7,8]. Templating strategies have emerged as a promising approach to direct nanoparticle growth and organization, which enables the formation of well-defined nanostructures with tailored properties.

**Scheme 1:**
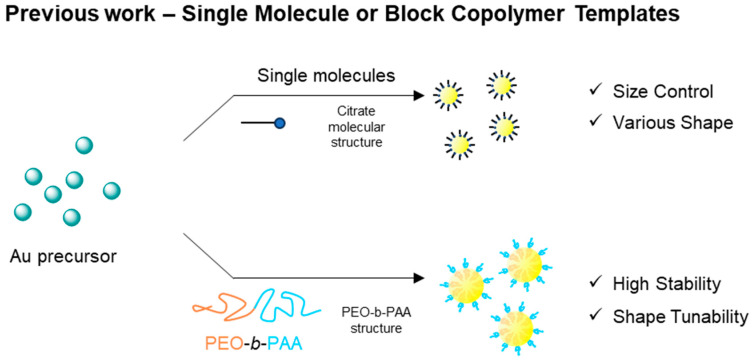
Synthetic approaches to Au NPs using small molecules or block copolymers as templates.

Conventional templating methods often employ small organic molecules or block copolymers that act as stabilizers, capping agents, or structural frameworks during nanoparticle synthesis (Scheme 1). For instance, small molecules such as citric acid have been successfully used to produce spherical Au NPs with tunable sizes and shapes—including cubes and prisms—via seed-mediated growth methods [9,10,11]. Block copolymers offer further advantages in stability and shape control by leveraging their intrinsic self-assembly behavior [12,13]. The molecular weight and structure of block copolymers can influence nanoparticle morphology, while their surface interactions help preserve and enhance the optical and electronic properties of nanoparticles. Recent progress in synthesizing block copolymers with uniform molecular weight distributions has further highlighted their potential as precision templates for nanostructure formation [14,15,16,17].

Despite the success of these approaches, challenges persist in achieving hierarchical and interconnected nanostructures with controlled morphology and assembly. Au nanonetwork structures exhibit distinct collective properties, including enhanced plasmonic coupling, charge transport, and structural stability, which are advantageous for applications in sensing, photonics, and catalysis [4,5,6,8]. Compared to isolated nanoparticles, their interconnected morphology enables synergistic functionalities. However, despite this potential, precise control over the assembly and morphology of Au nanonetworks remains limited, with relatively few systematic studies exploring supramolecular templating strategies to achieve such architectures. Supramolecular polymers have recently emerged as an alternative class of materials capable of addressing these limitations [18,19,20]. These polymers are constructed through dynamic, non-covalent interactions such as hydrogen bonding, π–π stacking, and hydrophobic forces. The reversible nature of these interactions enables the formation of tunable morphologies—including fibrous, branched, and interconnected network architectures—that are often difficult to access using conventional polymer templates. In addition, the use of functionalized building blocks allows supramolecular polymers to act as highly versatile templates for nanoparticle synthesis, which offers new opportunities for designing complex and functional nanostructures [21].

Among various supramolecular building blocks, benzene-1,3,5-tricarboxamide (BTA) derivatives have gained prominence due to their ability to form well-defined supramolecular assemblies [19,22]. BTA-based systems exhibit strong directional hydrogen bonding and hydrophobic interactions, which promote the formation of hierarchical structures in solutions [23]. Functionalizing the BTA core with tailored side groups provides additional control over the assembly process and allows the design of networked nanostructures suitable for nanoparticle templating [20]. However, achieving precise control over nanoparticle morphology through supramolecular templates remains challenging due to the dynamic nature of non-covalent interactions and the sensitivity of supramolecular assemblies to external conditions. Parameters such as molecular dispersity, oligomer length, concentration, and environmental conditions (e.g., pH, temperature, and ionic strength) significantly influence the structure of the resulting nanoparticles [24]. However, the specific role of molecular dispersity in supramolecular templating remains underexplored, leaving a critical gap in understanding how molecular uniformity affects the supramolecular assembly and its ability to direct nanoparticle formation.

**Scheme 2:**
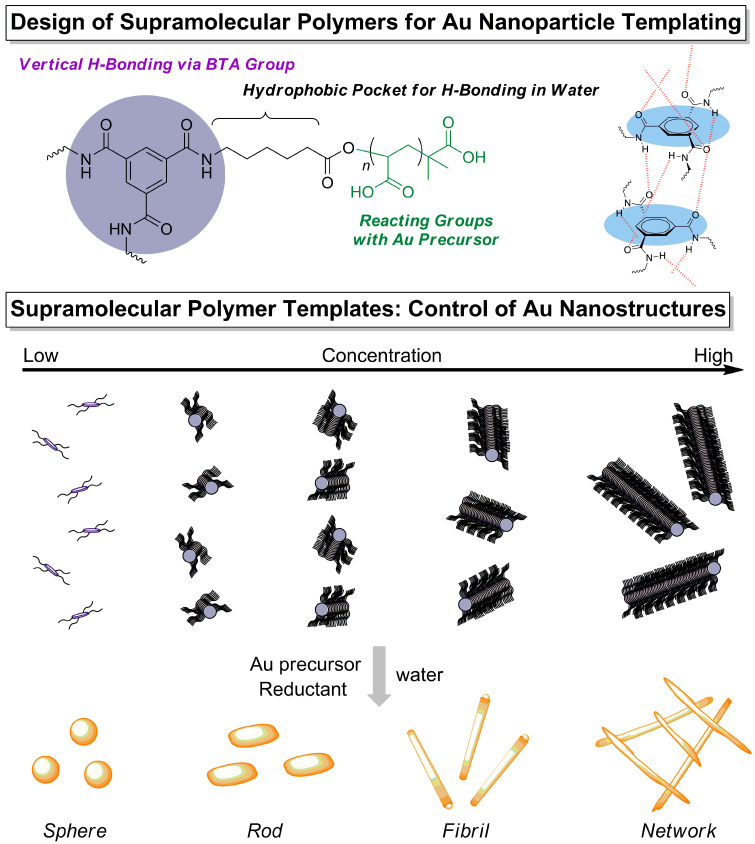
Schematic illustration of (top) the molecular design of BTA-oligo(AA pentamer)_3_ derivatives, featuring functional groups that promote supramolecular self-assembly through hydrogen bonding and hydrophobic interactions and facilitate nanostructure formation; and (bottom) the concentration-dependent control of nanostructures using supramolecular polymers as templates for nanoparticle synthesis.

In this study, we address the challenges of controlled nanoparticle templating by investigating BTA-oligo(AA)_3_-based supramolecular polymers as templates for the synthesis of gold nanostructures (Scheme 2). Specifically, we examine how molecular dispersity and concentration influence the formation of Au nanoparticles and nanonetworks in aqueous environments. In our previous report, we highlighted the importance of understanding the interaction between acetate groups and the Au precursor (HAuCl_4_) in directing the growth of Au NPs using double hydrophilic block copolymer templates [25]. Although the stoichiometry between sodium acetate and gold precursor was estimated to be 2:1 (one gold precursor per two sodium acetate molecules) based on Job’s plot analysis, controlled synthesis was achieved when three sodium acetate groups coordinated with one gold precursor. This deviation was attributed to the steric hindrance and electrostatic repulsion among carboxylate groups along the polymer backbone.

Thus, in this paper, we designed acrylic acid pentamers bearing an additional carboxylic acid at the initiator end, which provides six potential coordination sites for Au precursors. This design was incorporated into both polydisperse (BTA-[disperse AA pentamer]_3_) and discrete (BTA-[discrete AA pentamer]_3_) supramolecular templates to promote the controlled formation of Au nanostructures. This study demonstrates that discrete BTA-oligo(AA)_3_ oligomers with uniform molecular weight facilitate the formation of interconnected nanonetworks, whereas polydisperse oligomers favor the growth of isolated nanoparticles. By combining UV-vis spectroscopy, transmission electron microscopy (TEM), and dynamic light scattering (DLS) analyses, we clarify the role of supramolecular assembly in directing nanoparticle morphology.

These findings highlight the critical role of molecular dispersity in supramolecular templating and offer a pathway for designing hierarchical and interconnected nanostructures with controlled properties. This work contributes to the broader understanding of supramolecular polymer templates as a versatile platform for nanoparticle synthesis and highlights their potential application in nanotechnology, optoelectronics, and catalysis.

## 2. Experimental

**Materials.** *tert*-Butyl acrylate (*t*-BA, Sigma-Aldrich; St. Louis, MO, USA; 98%), *tert*-butyl α-bromoisobutyrate (Sigma-Aldrich; St. Louis, MO, USA; >98.0%), Cu(I)Br (Sigma-Aldrich; St. Louis, MO, USA; 99.999% trace metal basis), *N*,*N*,*N′*,*N″*,*N″*-pentamethyldiethylenetriamine (PMDETA, Sigma-Aldrich; St. Louis, MO, USA; 99%), 1,3,5-benzenetricarbonyl trichloride (Sigma-Aldrich; St. Louis, MO, USA; 98%), 6-aminocaproic acid (Sigma-Aldrich; St. Louis, MO, USA; >99%), dichloromethane anhydrous (DCM, Sigma-Aldrich; anhydrous, St. Louis, MO, USA; ≥99.8%), dimethyl sulfoxide (DMSO, Wako; Osaka, Japan; 99%), *N*,*N*-dimethylformamide (DMF, Sigma-Aldrich; St. Louis, MO, USA; 99.8%), 1,8-diazabicyclo[5.4.0]undec-7-ene (DBU, Sigma-Aldrich; St. Louis, MO, USA; ≥99%), trifluoroacetic acid (TFA, Sigma-Aldrich; St. Louis, MO, USA; *ReagentPlus^®^*, 99%), sodium hydroxide (Sigma-Aldrich; St. Louis, MO, USA; ≥98%), hydrochloric acid (Sigma-Aldrich; St. Louis, MO, USA; ≥37%), acetonitrile (Wako; Osaka, Japan; ≥99.9%), methanol (Wako; Osaka, Japan; ≥99.5%), diethyl ether, ethyl acetate, and hexane (Samchun Chemicals; Seoul, Republic of Korea; 99%) were used, as received from the manufacturers. Acetone (Sigma-Aldrich; ACS reagent, St. Louis, MO, USA; ≥99.5%) was purified by passing it through purification columns (JCM, JCM-3SPS-SA-6) and bubbling with dry nitrogen gas for more than 15 min immediately before use. Deuterated dimethyl sulfoxide (DMSO-*d*_6_, ≥99.9%) and deuterated chloroform (CDCl_3_, 99.8%) were purchased from Cambridge Isotope Laboratories, Inc. (Tewksbury, MA, USA).

**Synthesis of oligo(*t*-BA) (disperse/discrete oligo(*t*-BA)).** Oligo(*t*-BA) was synthesized and fractionated according to procedures previously reported in the literature. The degree of polymerization (*DP*_n_) of oligo(*t*-BA) was targeted to be five in this study [26].

**Synthesis of 6,6′,6″-((benzene-1,3,5-tricarbonyl)tris(azanediyl))trihexanoic acid.** The target molecule was prepared via the Schotten–Baumann reaction. A solution of NaOH (1N) was added to 6-aminohexanoic acid (3.3 equiv.) at room temperature. The mixture was stirred until complete dissolution, followed by cooling to 0–5 °C. A solution of 1,3,5-benzene tricarbonyl trichloride (1.0 equiv.) in DCM was then added dropwise to the cooled mixture. After 30 min, the reaction was allowed to warm to room temperature and stirred for an additional four hours. Subsequently, DCM was removed under pressure, and the resulting mixture was acidified with ice-cold 1N HCl. The white precipitate was collected by filtration, washed with ice-cold water, dried in a vacuum oven, and used directly in the subsequent reaction.

**General procedure for the synthesis of the three-armed star oligomer (BTA-oligo(*t*-BA)_3_).** To a solution of 6,6′,6″-((benzene-1,3,5-tricarbonyl)tris(azanediyl))trihexanoic acid (1.0 equiv.) in DMF was added oligo(*t*-BA)_3_ (vacuum dried, 3.6 equiv.) and DBU (3.6 equiv.) under an inert atmosphere. The mixture was stirred at room temperature for four hours. After completion, DMF was removed under reduced pressure, and the concentrated mixture was diluted with ethyl acetate. The organic phase was washed sequentially with ice-cold 1N HCl and brine, then dried over anhydrous Na_2_SO_4_. Removal of the organic solvent under reduced pressure yielded a colorless oily residue, which was purified via column chromatography to afford the target compound BTA-oligo(*t*-BA)_3_ as a colorless oil.

**General procedure for the deprotection of three-armed star oligomer (BTA-oligo(*t*-BA)_3_).** The BTA-oligo(*t*-BA)_3_ (1.0 equiv.) was dissolved in anhydrous DCM under Ar. Trifluoroacetic acid (30.0 equiv.) was added dropwise to this clear solution. The mixture was allowed to stir at room temperature for 24 h, after which volatiles were removed under reduced pressure. The obtained residue was dissolved in a minimum amount of MeOH. The addition of DCM (large excess) to this solution resulted in white precipitates. After this product had been filtered, washed with cold DCM, and dried under vacuum, the target compound BTA-oligo(acrylic acid, AA)_3_ was obtained.

**Supramolecular self-assembly behavior.** To investigate the self-assembly behavior of BTA-oligo(AA)_3_ in aqueous media, samples of BTA-oligo(AA)_3_ with a concentration ranging from 0.1 mg/mL to 1.0 mg/mL were prepared. After the filtration of the prepared solutions, the self-assembly behavior of BTA-oligo(AA)_3_ and the corresponding aggregation were analyzed using a UV-vis spectrometer and dynamic light scattering (DLS).

**Synthesis of Au nanostructures via supramolecular polymer templating.** Au NPs were synthesized using supramolecular polymer templates according to the following procedure. As a representative example, BTA-oligo(AA)_3_ (1.9 mg, 0.018 mmol of carboxylic acid groups in the oligo(AA)_3_ block) was dissolved in 1.9 mL of deionized water, followed by the addition of 0.036 mL of 1.0 M NaOH (0.036 mmol, 2.0 equiv. per carboxylic acid group). To this solution, 0.018 mL of HAuCl_4_·2H_2_O (0.5 M, 0.009 mmol) was added, and the mixture was stirred for 5 min. Subsequently, 0.036 mL of 0.5 M ascorbic acid (0.018 mmol) was added. After three minutes of vigorous stirring, a visible color change was observed, which indicates nanoparticle formation. The resulting solution was dialyzed against deionized water using a dialysis membrane (MWCO 12,000–14,000, SpectraPore, Raleigh, NC, USA) to remove residual reagents and byproducts.

**Measurements.** The number-average molecular weight (*M*_n_) and dispersity (*M*_w_/*M*_n_) of the polymers were measured via size-exclusion chromatography (SEC) at 25 °C using THF as the eluent. THF-SEC was performed with three polystyrene-gel columns [LF-404 (from Shodex, Tokyo, Japan); pore size, 150 Å; 8 mm i.d. × 300 mm, LF-404 (from Shodex); pore size, 500 Å; 8 mm i.d. × 300 mm, LF-404 (from Shodex); pore size, 1500 Å; 8 mm i.d. × 300 mm] were connected to a PU-4180 pump, a RI-4035 refractive-index detector, and a UV-4075 ultraviolet detector (JASCO, Randburg, South Africa). The flow rate was set to 0.3 mL min^−1^. The columns were calibrated against eight standard polystyrene samples (Shodex; *M*_p_ = 1160–676,000; *M*_w_/*M*_n_ = 1.03–1.11) to analyze the oligomer samples. ^1^H nuclear magnetic resonance (NMR) spectra were recorded in ppm using an Ultrashield spectrometer (Bruker, Billerica, MA, USA) operating at 300 MHz. Spectra were recorded in deuterated solvents (CDCl_3_, DMSO-*d*_6_) at room temperature. Mass spectra of the prepared oligomers/polymers were acquired as Na^+^ adducts (*m*/*z*) using a Shimadzu MALDI-TOF (Axima Confidence, Berryville, VA, USA) mass spectrometer.

UV-Vis absorption spectra were recorded using a JASCO V-770 UV-visible/NIR spectrophotometer (JASCO, Randburg, South Africa). A halogen lamp with a wavelength range of 190–2700 nm and wavelength accuracy of ±0.3 nm (at 656.1 nm) served as the light source.

The hydrodynamic diameter of the supramolecular assemblies formed by BTA-oligo(AA)_3_ was measured using a Malvern Zeta Sizer Nano ZS90 particle analyzer (Malvern Panalytical, Malvern, UK). A He-Ne laser at 633 nm with a maximum power of 5 mW was used as a light source, and scattered light was collected at an angle of 90°. The temperature was maintained at 25 °C throughout all measurements. The translational diffusion coefficient and hydrodynamic radius (*R*_h_) of the aggregates were calculated using autocorrelation analysis of the scattered light intensity over time. *R*_d_ values are expressed as mean ± SD (*n* = 3). The morphology, size, and size distribution of the prepared NPs were examined using transmission electron microscopy (TEM, Tecnai FEI T20, and JEOL JEM-2100, JEOL, Tokyo, Japan) operated at 200 kV and equipped with a Gatan CCD camera (Gatan, Inc., Pleasanton, CA, USA).

## 3. Results and Discussion

**Syntheses and characterization of BTA-oligo(acrylic acid pentamer)_3_ series.** The Meijer group has reported extensively on the aggregation and phase behavior of *N*,*N*′,*N*′′-trialkyl-benzene-1,3,5-tricarboxamides (BTAs) [22,27,28,29]. In particular, they demonstrated that BTAs incorporating both hydrophobic aliphatic chains and hydrophilic PEO groups interact in aqueous solutions in a concentration-dependent manner, which enables control over supramolecular structures resembling nanoscale fibers. They also showed that different fiber morphologies—such as twisted, thin linear, and slightly compressed forms—can be achieved by molecular modifications, including variations in the number of hydroxyl groups or chirality.

Based on these findings, we focused on the role of functional molecular design in directing the formation of linear supramolecular assemblies. The oligo(ethylene oxide) (OEO) covalently attached to the BTA core, for instance, is a functional building block that exhibits exceptional stability in an aqueous solution and still maintains the integrity of linear supramolecular configurations. Replacing OEO with alternative functional groups offers a strategy to induce significant structural transitions. For example, the incorporation of acrylic acid (a functional group capable of forming intermolecular hydrogen bonds or electrostatic interactions with external ionic precursors) promotes the self-assembly of supramolecular architectures into more complex or non-linear configurations, thus broadening the range of accessible structural motifs.

**Figure 1:**
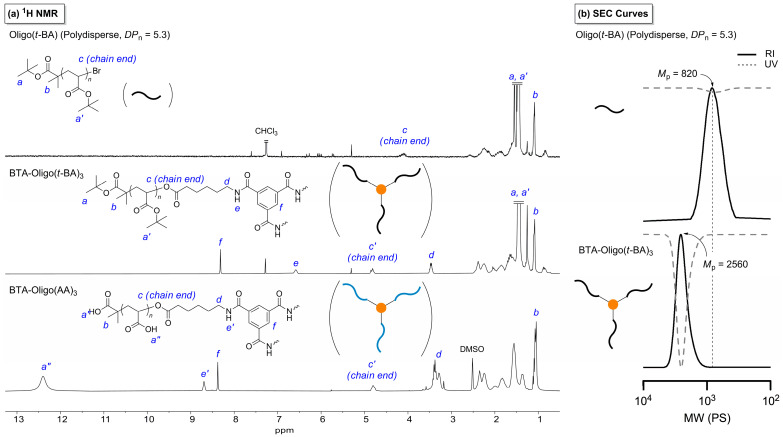
Structural analysis of oligo(*t*-BA) based products. (**a**) ^1^H NMR spectra of disperse oligo(*t*-BA), disperse BTA-oligo(*t*-BA)_3_, and disperse BTA-oligo(AA)_3_ in CDCl_3_ or DMSO and (**b**) the corresponding SEC curves of disperse oligo(*t*-BA) and BTA-oligo(*t*-BA)_3_ calibrated using polystyrene (PS) standards in DMF.

In order to explore the impact of functional groups on the supramolecular behavior of BTA-based molecules, a series of BTA supramolecules incorporating acrylic acid-functionalized arm chains were synthesized using the procedure outlined in Scheme S1. Initially, 6-aminocaproic acid was used to introduce hydrophobic aliphatic chains into 1,3,5-benzenetricarbonyl trichloride through an amidification reaction. The successful incorporation of the amine, aliphatic, and carboxylic acid groups of 6-aminocaproic acid into benzoyl chloride was confirmed via ^1^H NMR and ^13^C NMR spectroscopy (Figures S1 and S2). Simultaneously, oligomeric *tert*-butyl acrylate (oligo(*t*-BA)) with a targeted *DP*_n_ = 5 was synthesized via atom transfer radical polymerization (Figure 1a, top). The structural characterization of the synthesized oligo(*t*-BA) was confirmed using ^1^H NMR spectroscopy, where a characteristic resonance at 4.12 ppm (*c*) was assigned to the -C*H*-Br proton at the oligomer chain terminus.

In addition, the *DP*_n_ of disperse oligo(*t*-BA) was calculated from the integration ratio of two methyl groups in the initiator unit at 1.15 ppm (*b*) to the *tert*-butyl groups at 1.42 ppm (*a* and *a’*), which enabled the estimation of an average *DP*_n_ of 5.30. The oligo(*t*-BA) chains were subsequently conjugated to the terminal positions of 6,6′,6″-((benzene-1,3,5-tricarbonyl)tris(azanediyl))trihexanoic acid under ambient conditions, which led to the formation of BTA-oligo(*t*-BA)_3_ molecules. The incorporation of the acrylate moiety was verified using ^1^H NMR spectroscopy, which revealed characteristic signals of the acrylate group and confirmed its stable attachment (Figure 1a, middle). For example, the ^1^H NMR peaks derived from the aromatic group in the BTA core can be clearly observed (*f*, 8.30 ppm), including those for the chain ends of oligo(*t*-BA) (*c’*, 4.76 ppm), *tert*-butyl groups (*a* and *a’*, 1.42 ppm), and amine protons (*e*, 6.56 ppm).

SEC analysis further revealed an approximate threefold increase in molecular weight after the coupling reaction, and UV absorption at 270 nm overlapped with the refractive index (RI) signal, which confirms the successful attachment of oligo(*t*-BA) chains to the BTA core (Figure 1b; *M*_p, oligo(*t*-BA)_ = 820 and *M*_p, BTA-oligo(*t*-BA)3_ = 2560). Subsequent deprotection of the *tert*-butyl groups was carried out using trifluoroacetic acid (TFA), yielding BTA derivatives bearing multiple terminal acrylic acid groups. The disappearance of the tert-butyl proton signal at approximately 1.4 ppm in the ^1^H NMR spectrum (Figure 1a, bottom) confirmed the deprotection. Finally, the synthesized product was purified by prolonged dialysis in deionized water to remove residual reactants and byproducts, which resulted in high-purity BTA derivatives suitable for subsequent characterization and application.

**MALDI analysis of the polydisperse *t*-BA oligomer and isolated discrete *t*-BA oligomer.** Recent advances in polymer chemistry have emphasized the critical role of molecular dispersity in determining the unique properties of oligomers and polymers. For instance, the Hawker group reported that acrylic acid-based polymers exhibit notable differences in their properties depending on their polydispersity index [30]. Similarly, other studies have shown that brush-like polymers with a dispersity of *Ð* = 1 display distinct structural features and enhanced performance, which underlines the importance of molecular uniformity [14,31]. These findings demonstrate that dispersity serves as a powerful parameter for tuning material behavior, even when the molecular composition remains unchanged.

Inspired by these insights, we explored the use of oligo(*t*-BA) with defined repeating units to investigate its impact on the supramolecular behavior of BTA-based materials. Polydisperse *t*-BA oligomer (*DP*_n_ = 5.30 and *Ð* = 1.20) was initially synthesized via atom transfer radical polymerization. The oligomer mixture was characterized using matrix-assisted laser desorption/ionization time-of-flight mass spectroscopy (MALDI-ToF-MS) to assess the molecular composition and structural uniformity of the oligomers.

**Figure 2:**
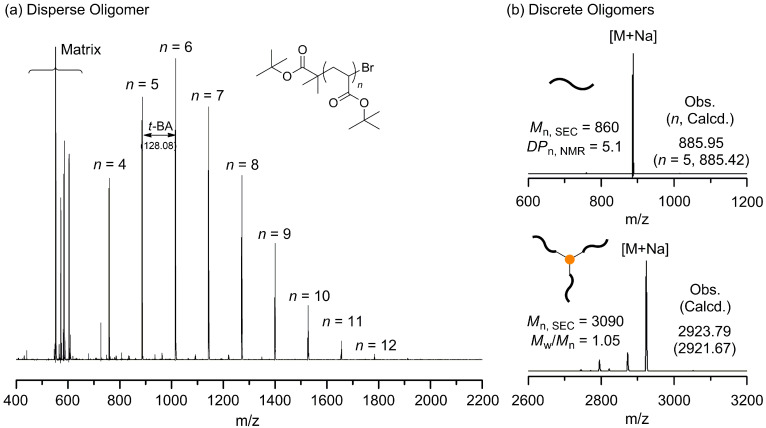
Representative MALDI-ToF-MS spectra of oligo(*tert*-butyl acrylate) (oligo(*t*-BA)). (**a**) MALDI-ToF-MS spectrum of the crude polydisperse oligo(*t*-BA) sample. (**b**) MALDI-ToF-MS spectrum of the discrete oligo(*t*-BA) (top) and BTA-oligo(*t*-BA)_3_ (bottom).

MALDI-ToF-MS analysis of the polydisperse oligomer revealed distinct molecular ion peaks with a consistent mass interval corresponding to the molecular weight of the *t*-BA monomer (128.08). This result verifies the presence of a well-defined oligomeric series within the dispersed sample (Figure 2a). Using a column chromatography method capable of separating the individual oligomer species present in the polydisperse oligomer, we successfully isolated discrete oligomers with defined numbers of repeating units (most notably the pentamer) from the polydisperse *t*-BA oligomers. The purified fractions exhibited a single, well-resolved molecular ion peak in MALDI-ToF-MS, which confirms the successful separation and molecular precision of the discrete oligomers (Figure 2b top and Figure S3 top; observed *m*/*z*: 885.95 for discrete pentamer; [5 *t*-BA units + initiator unit + Na^+^]; *DP*_n, NMR_ = 5.10).

For a comparative study with the previously synthesized BTA-oligo(AA)_3_ containing polydisperse units, a discrete BTA-oligo(*t*-BA)_3_ was synthesized using a discrete oligomer with precisely five *t*-BA repeating units (Figure 2b bottom, Figure S3 middle, and Figure S4; observed *m*/*z*: 2923.79 for BTA-oligo(*t*-BA)_3_; [15 *t*-BA units + 3 initiator unit + BTA core + Na^+^]). Subsequently, deprotection was performed to obtain the final product, discrete BTA-oligo(AA)_3_ (Figure S3 bottom). The resulting materials were designated as discrete BTA-oligo(t-BA)_3_ and discrete BTA-oligo(AA)_3_, respectively, to emphasize their defined molecular structure and uniform dispersity.

**Supramolecular behavior of self-assembled BTA-oligo(AA)_3_.** To examine whether substituting the ethylene oxide arms of BTA molecules with acrylic acid affects their self-assembly behavior, we investigated the supramolecular organization of BTA-oligo(AA)_3_ in aqueous solution. We first assessed the self-assembly behavior of polydisperse BTA-oligo(AA)_3_ in water by analyzing its concentration-dependent behavior using UV-Vis spectroscopy.

**Figure 3:**
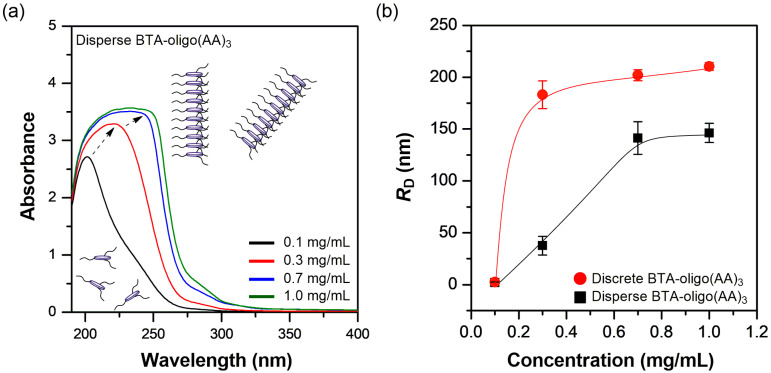
(**a**) UV-Vis spectra for different concentrations of polydisperse BTA-oligo(AA)_3_ in aqueous solution and (**b**) DLS analysis of discrete and polydisperse BTA-oligo(AA)_3_ at various concentrations.

At a concentration of 0.1 mg/mL in an aqueous solution, polydisperse BTA-oligo(AA)_3_ exhibited a single absorption peak at approximately 201 nm (Figure 3a). This result suggests that the molecules remain molecularly dispersed in a solution, with minimal intermolecular interactions. In contrast, at a concentration of 0.3 mg/mL, the maximum absorption peak shifts to 221 nm. It is well established that π–π stacking interactions among BTA derivatives lead to a red shift in the absorption maximum [32]. Therefore, the absorption at 221 nm observed for polydisperse BTA-oligo(AA)_3_ can be attributed to such stacking interactions in an aqueous solution. As the concentration of polydisperse BTA-oligo(AA)_3_ increases, a broad absorption band gradually emerges between approximately 220 nm and 250 nm, which is a particularly noteworthy feature. A similar trend is observed for the discrete BTA-oligo(AA)_3_ system, which exhibits comparable concentration-dependent absorbance behavior (Figure S5), further confirming the self-assembly characteristics of both supramolecular systems. Previous studies have shown that BTA derivatives containing PEG chains, hydroxyl groups, or hydrophobic aliphatic substituents typically exhibit two distinct absorption peaks as the concentration increases, with the lower wavelength peak at 211 nm becoming more prominent [27,32]. However, in the case of polydisperse BTA-oligo(AA)_3_, a broad absorption band appears within the 220 nm to 250 nm range as the concentration increases. This observation suggests that as concentration increases, the molecules not only form stacked structures but also adopt a different structural arrangement compared to previously reported BTA derivatives.

In general, changes in the concentration of BTA derivatives have been reported to induce self-assembly in a solution, which not only leads to an increase in hydrodynamic volume but also impacts the formation of supramolecular structures. For example, the BTA derivative functionalized with PEG exhibits a hydrodynamic diameter of ten nanometers in an aqueous solution at a concentration of 3.0 mg/mL [33]. This significant increase in hydrodynamic volume provides important evidence that supports the formation of supramolecular structures. In this study, disperse BTA-oligo(AA)_3_ exhibited an average *R*_D_ value of 2.05 nm at a concentration of 0.1 mg/mL (Figure 3b, black dot). This result indicates that at 0.1 mg/mL, the synthesized oligomer forms minimal self-assembled structures, such as stacked assemblies. These findings are consistent with the UV-vis results. However, at a concentration of 0.3 mg/mL, the *R*_D_ of polydisperse BTA-oligo(AA)_3_ increased to 37.58 nm, which indicates that the oligomer concentration significantly affects the hydrodynamic volume. This volume increase suggests the onset of self-assembly processes. Moreover, at concentrations of 0.7 mg/mL and 1.0 mg/mL, *R*_D_ further increased to 141.30 nm and 146.24 nm, respectively. This clearly demonstrates that polydisperse BTA-oligo(AA)_3_ has distinct supramolecular characteristics at higher concentrations.

At a concentration of 0.1 mg/mL, discrete BTA-oligo(AA)_3_ exhibits an *R*_D_ value of 2.45 nm. This means that, similar to polydisperse BTA-oligo(AA)_3_, the discrete BTA-oligo(AA)_3_ solution at low concentrations moves freely rather than undergoing self-assembly between oligomers. At 0.3 mg/mL, the oligomer displays an *R*_D_ value of 183.10 nm, which indicates that an assembled structure formed through intermolecular interactions, showing a similar trend to that of polydisperse BTA-oligo(AA)_3_ at the same concentration. Notably, the oligomer synthesized in this study exhibited a relatively high *R*_D_ value even at a low concentration (0.3 mg/mL). This behavior indicates the rapid formation of supramolecular structures, even at low concentrations. Furthermore, the increase in *R*_D_ remained modest as the concentration was raised to 1.0 mg/mL, which indicates a limited change in aggregate size over this range. We cautiously propose that the resulting supramolecular assemblies may adopt relatively complex architectures. To gain further insights into the structural features of BTA-oligo(AA)_3_, we conducted additional analyses using electron microscopy.

**Figure 4:**
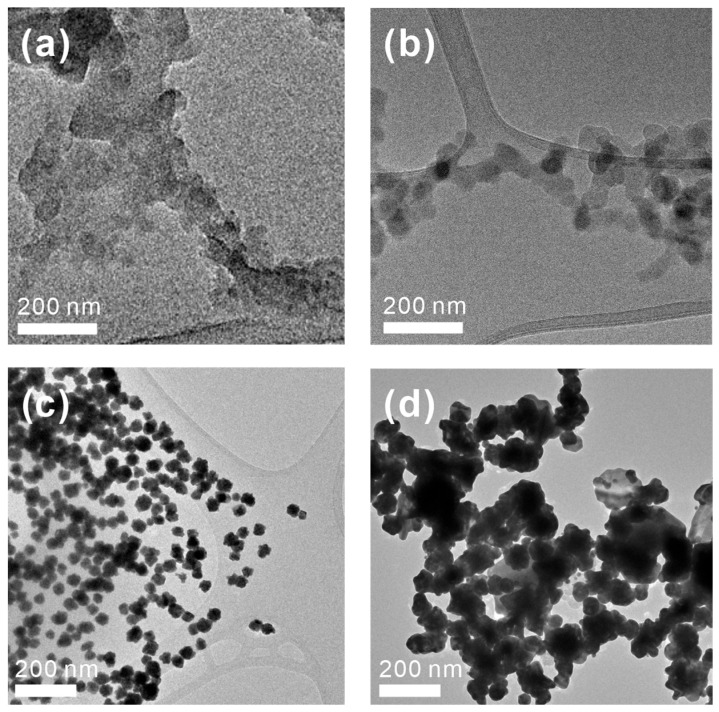
TEM images of self-assembled (**a**) disperse BTA-oligo(AA)_3_ and (**b**) discrete BTA-oligo(AA)_3_. Corresponding Au nanostructures synthesized using (**c**) disperse BTA-oligo(AA)_3_ and (**d**) disperse BTA-oligo(AA)_3_.

Structural Characterization and Dispersity Effects on the Formation of Supramolecular Polymer Networks via BTA-oligo(AA)_3_ Assemblies. To investigate the structural features of BTA-oligo(AA)_3_ assemblies in greater detail, we examined their morphologies using transmission electron microscopy (Figure 4). Two distinct types of supramolecular structures were observed, which correspond to the polydisperse and discrete BTA-oligo(AA)_3_ systems. Figure 4a presents the supramolecular structure of polydisperse BTA-oligo(AA)_3_, which lacks distinctive repetitive shapes or regular organization. However, it was difficult to define a specific structural conformation for the polydisperse BTA-oligo(AA)_3_. In contrast, the discrete BTA-oligo(AA)_3_ formed a well-connected polymeric network (Figure 4b). This network exhibited a thickness in the order of tens of nanometers, in stark contrast to previously reported BTA-based nanofibers, which typically assemble into structures with thicknesses of only a few nanometers.

The structure of the synthesized BTA-oligo(AA)_3_ shares a similar core configuration with previously reported BTA derivatives [22] but is surrounded by acrylic acid groups. Furthermore, this configuration facilitates the formation of supramolecular assemblies through hydrogen bonding and π–π interactions within the BTA core and also enables additional bonding interactions through the carbonyl and hydroxyl groups of the acrylic acid. Specifically, intermolecular forces between acrylic acid units and interactions between the BTA core and acrylic acid groups are anticipated to drive the formation of a complex supramolecular structure that is distinct from previously reported linear assemblies. These varied interactions are expected to contribute significantly to the formation of supramolecular assemblies with lengths extending to tens of nanometers. Furthermore, these interactions likely promote a multidirectional rather than unidirectional arrangement of the supramolecular polymers, which leads to a more intricate three-dimensional structure.

Based on these findings, the two types of synthesized BTA-oligo(AA)_3_ confirm the ability to form complex, network-like structures. More specifically, hydrogen bonding and π–π interactions within the BTA core, combined with additional intermolecular interactions from the functionalized acrylic acid groups, facilitate the formation of these network architectures. Variations in oligomer dispersity further affect the regularity of the polymer networks. This suggests that dispersity is indeed a key factor in determining structural consistency.

**Synthesis and Analysis of Au Nanostructures Using Various BTA-oligo(AA)_3_ Templates.** We observed that discrete BTA-oligo(AA)_3_-based supramolecular structures exhibit distinct configurations in aqueous solution, depending on their molecular weight and dispersity. These structures were then employed as templates to precisely control the formation of metal nanostructures.

The synthesized BTA-oligo(AA)_3_ exhibits strong potential for applications in aqueous environments due to the presence of acrylic acid groups surrounding the BTA core. The acrylate functionality allows for ionic or coordinative bonding with metal precursors, which makes BTA-oligo(AA)_3_ a promising template for synthesizing various metal and metal oxide nanostructures. For instance, PEO-*b*-PAA block copolymers have been reported to form spherical gold nanoparticles by creating micellar structures with gold precursors [25,34,35], while PS-*b*-PAA-*b*-PS triblock copolymers have enabled the formation of hollow gold nanoparticles [36,37,38]. Similarly, cellulose-*g*-PAA-*b*-PS copolymers used as nanoreactors have facilitated the synthesis of metal nanorods with lengths in the hundreds of nanometers and metal oxides extending to micron-scale lengths [39]. Beyond these linear and unidirectional structures, BTA-oligo(AA)_3_ offers the potential to generate network-like metal nanostructures when used as a supramolecular nanoreactor. To explore this capability, we employed both polydisperse and discrete BTA-oligo(AA)_3_ as templates for synthesizing gold nanostructures and examined the resulting morphologies using TEM (Figure 4c,d).

Au nanostructures synthesized using polydisperse BTA-oligo(AA)_3_ display dimensions of several tens of nanometers and are observed as individually dispersed, single nanoparticles (Figure 4c). In aqueous solution, polydisperse BTA-oligo(AA)_3_ tends to form irregular supramolecular structures, which can lead to the formation of gold nanoparticles with varied and random sizes. Larger gold particles, potentially reaching micron-scale dimensions, precipitate out of solution due to low dispersibility and are therefore not observed in the electron microscopy images. As a result, Figure 4c only displays the smaller, well-dispersed gold nanoparticles that remain stably suspended in the solution.

In contrast to the gold nanoparticles formed using polydisperse molecular templates, those synthesized with discrete BTA-oligo(AA)_3_ exhibit a nanostructure resembling a connected network, characterized by interparticle connectivity, albeit with some degree of non-uniformity (Figure 4d). Notably, the thickness of the gold nanonetwork closely matches (or, in some regions, exceeds) the width of the individual polymer chains. This observation suggests that the supramolecular organization of discrete BTA-oligo(AA)_3_ retains sufficient stability to serve as an effective template, which enables the controlled assembly of networked gold nanostructures. The discrete BTA-oligo(AA)_3_ supramolecular structure, with lengths typically below 100 nm, is primarily stabilized through intermolecular forces such as hydrogen bonding and π–π interactions within the aromatic BTA core. Upon the addition of the gold precursor, additional coordination bonding occurs between the acrylate groups and the metal ions, which further facilitates the directed formation of metal nanoparticle structures. The Au nanostructures formed through these complex interactions showed lengths up to several hundred nanometers, which confirms the successful integration of gold nanoparticles into the pure supramolecular scaffold.

In line with the initial hypothesis that discrete BTA-oligo(AA)_3_ oligomers could form concentration-dependent supramolecular structures, we examined their influence on gold nanostructure formation at two concentrations: 0.1 mg/mL and 2.0 mg/mL (Figure S6). At 0.1 mg/mL, predominantly discrete spherical Au nanoparticles were observed, indicating that under dilute conditions, the oligomers act as individual molecular templates with minimal supramolecular assembly. At 2.0 mg/mL, network-like Au structures emerged, though with some non-uniformity in thickness and morphology. Importantly, despite these variations, the system exhibited a clear tendency to form interconnected architectures rather than aggregated clusters. These results underscore the pivotal role of concentration in modulating the supramolecular organization of discrete BTA-oligo(AA)_3_ and directing the morphology of templated gold nanostructures. Further studies exploring the roles of specific intermolecular interactions and the molecular weight of oligomers in directing metal nanostructure formation will be presented in future publications.

**Figure 5:**
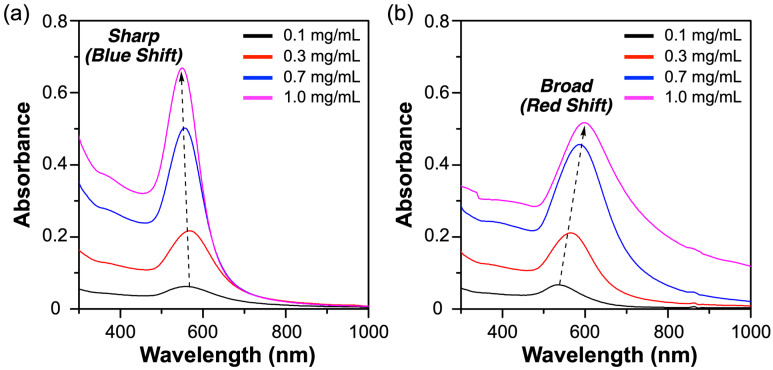
UV-Vis spectra of Au nanostructures synthesized using (**a**) polydisperse BTA-oligo(AA)_3_ and (**b**) discrete BTA-oligo(AA)_3_ as templates. For both the polydisperse and discrete BTA-oligo(AA)_3_ samples, concentrations were consistently set to 0.1, 0.3, 0.7, and 1.0 mg/mL to ensure comparability.

Additional UV-vis analyses were conducted to clearly elucidate the structural differences in the synthesized nanoparticles. For consistency and comparability, absorbance measurements of both polydisperse and discrete BTA-oligo(AA)_3_ samples were performed at concentrations of 0.1, 0.3, 0.7, and 1.0 mg/mL under identical conditions (Figure 5a). As anticipated, gold nanoparticles synthesized using polydisperse BTA-oligo(AA)_3_ exhibited a characteristic absorbance peak near 570 nm. With increasing concentration, a slight blue shift was observed, which indicates the formation of nanoparticles with a broad size distribution ranging from several nanometers to tens of nanometers.

In contrast, Au nanomaterials synthesized from discrete BTA-oligo(AA)_3_ showed an absorbance peak centered at 570 nm for a concentration of 0.1 mg/mL. This indicates the presence of predominantly dispersed individual Au nanoparticles at lower concentrations (black line in Figure 5b). As the concentration increased to 0.3 mg/mL, the maximum absorbance shifted to 580 nm, accompanied by a noticeable broadening of the absorbance band. At higher concentrations (specifically 0.7 mg/mL and 1.0 mg/mL), the maximum absorbance further red-shifted to 585 nm and 595 nm, respectively, with additional spectral features appearing beyond 700 nm. These findings indicate the progressive formation of aggregated nanostructures or interconnected networks at higher concentrations.

At a concentration of 1.0 mg/mL, the resulting gold nanomaterials exhibit a loosely defined network structure with limited uniformity. Although a distinct absorption peak is not observed in the long-wavelength region, the spectral features shown in Figure 5b indicate that the Au nanomaterials formed under these conditions possess interconnected structures. This behavior aligns with the network-like supramolecular organization observed for discrete BTA-oligo(AA)_3_ at higher concentrations and provides indirect evidence for the formation of a similarly networked morphology in the gold nanomaterials.

**Scheme 3:**
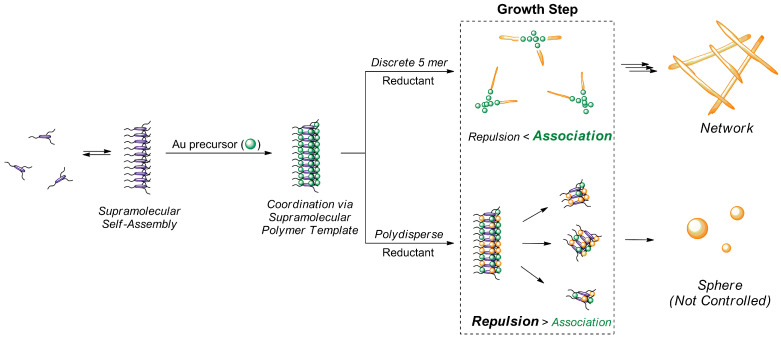
Schematic illustration of the proposed mechanism, illustrating the effect of the polymer dispersity on the formation of gold nanostructures.

**Proposed Mechanism of Au-Nanostructure Formation Based on Discrete Supramolecules.** The schematic representation in Scheme 3 illustrates the mechanism underlying the impact of polymer dispersity on the growth and resulting morphology of gold nanostructures. The process begins with the supramolecular self-assembly of discrete or polydisperse BTA-oligo(AA)_3_ molecules, forming supramolecular polymer templates in aqueous solution. Upon the introduction of a gold precursor, these templates facilitate the coordination and subsequent reduction of gold ions, which leads to the growth of nanostructures.

For discrete BTA-oligo(AA)_3_, the uniformity of discrete oligomers promotes the formation of well-ordered supramolecular polymer templates. These templates effectively balance repulsive and associative forces during the growth step, which favors an association-dominant mechanism. As a result, gold ions are deposited in a controlled manner along the supramolecular template, which leads to the formation of a highly organized network structure. In contrast, polydisperse systems (characterized by variations in chain length and molecular weight) form heterogeneous templates with irregular structural features. During the nanoparticle growth process, repulsion between disordered polymer segments and reduced gold ions dominates, which leads to the formation of isolated, poorly controlled spherical nanoparticles rather than a cohesive network.

This proposed mechanism provides a clear explanation for the structural differences observed in gold nanostructures synthesized from discrete versus polydisperse oligomers. The ability of discrete BTA-oligo(AA)_3_ to direct the assembly of interconnected gold nanonetworks highlights its potential as a versatile and robust template for engineering nanostructures with tailored morphology and properties.

## 4. Conclusions

In this study, we investigated the impact of polymer dispersity and supramolecular assembly on the formation of gold nanostructures using discrete and polydisperse BTA-oligo(AA)_3_ systems as templates. Our findings revealed that the molecular uniformity of the discrete oligomer promoted the formation of well-defined supramolecular nanonetworks, which the controlled association of the gold precursor further stabilized. In contrast, the polydisperse BTA-oligo(AA)_3_ system, characterized by heterogeneity in chain length, led to less ordered assemblies and the formation of spherical gold nanoparticles with limited control over size and morphology.

Spectroscopic and microscopic analyses demonstrated that the discrete system included red-shifted plasmonic behavior, consistent with nanonetwork formation, particularly at higher concentrations. This behavior was supported by UV-Vis and TEM analyses, which confirmed distinct structural differences between the discrete and polydisperse systems. The proposed mechanism highlights the critical role of supramolecular polymer templates in coordinating precursor molecules and guiding the growth of nanostructures through a balance of repulsive and associative interactions. These findings advance our understanding of how molecular dispersity affects nanostructure formation and provide a foundation for designing precision supramolecular templates for targeted nanomaterials synthesis. Future research will focus on tailoring supramolecular nanostructures through controlled variations in oligomer molecular weight and functional group composition, as well as exploring hybrid systems that combine discrete and polydisperse oligomers to access nanostructures with tunable properties.

## Data Availability

The original contributions presented in this study are included in the article/Supplementary Materials. Further inquiries can be directed to the corresponding authors.

## Supplementary Materials

Supplementary data to this article can be found online at: https://www.mdpi.com/article/10.3390/polym17121662/s1. Scheme S1: Synthetic procedure for the three-armed BTA-oligo(AA)_3_ molecule; Figure S1: ^1^H NMR spectrum of 6,6′,6″-((benzene-1,3,5-tricarbonyl)tris(azanediyl))trihexanoic acid; Figure S2: ^13^C NMR spectrum of 6,6′,6″-((benzene-1,3,5-tricarbonyl)tris(azanediyl))trihexanoic acid; Figure S3: ^1^H NMR spectra of (a) discrete oligo(*t*-BA) with *DP*_n_ = 5, and (b) discrete BTA-oligo(*t*-BA)_3_ and (c) discrete BTA-oligo(AA)_3_ synthesized using discrete oligo(*t*-BA) with *DP*_n_ = 5; Figure S4: SEC curves for discrete oligo(*t*-BA) and its corresponding discrete BTA-oligo(*t*-BA)_3_; Figure S5: UV-vis absorption spectra of discrete BTA-oligo(AA)_3_ at different concentrations (0.1, 0.3, 0.7, and 1.0 mg/mL), showing the effect of concentration on optical behavior; Figure S6: TEM images showing structural variations in gold nanomaterials synthesized using discrete BTA-oligo(AA)_3_ templates. The images illustrate the effect of precursor and oligomer concentrations on nanostructure formation. The concentrations of discrete BTA-oligo(AA)_3_ templates in aqueous solution are: (a) 0.1 mg/mL, (b) 1.0 mg/mL, and (c) 2.0 mg/mL.
