# Supplementary material for: Controlled Formation of Au Nanonetworks via Discrete BTA-Oligo(Acrylic Acid)3 Supramolecular Templates
Source: Polymers (Basel). 2025 Jun 15;17(12):1662. doi: 10.3390/polym17121662 (PMC12196678; doi:10.3390/polym17121662)
Supplement: Supplementary file 1 [file polymers-17-01662-s001.zip › polymers-3695243-supplementary.pdf]

Controlled Formation of Au Nanonetworks via Discrete BTA-Oligo(Acrylic Acid)3 Supramolecular Templates

Sadaf Aiman 1,2,†, Soonyoung Choi 1,†, Hyosun Lee 3, Sang-Ho Lee 1,4, and Eunyong Seo 1,5,**

[1]Center for Specialty Chemicals, Korea Research Institute of Chemical Technology, Ulsan 44412, Republic of Korea

[2]Department of Chemistry, School of Natural Sciences, National University of Sciences and Technology, Is-lamabad 44000, Pakistan

[3]Department of Chemistry and Green-Nano Materials Research Center, Kyungpook National University, 80 Daehakro, Bukgu, Daegu 41566, Republic of Korea

[4]Department of Chemical and Biochemical Engineering, Dongguk University, Seoul 04620, Republic of Korea

[5]Department of Chemical Engineering, Ulsan College, Ulsan 44610, Republic of Korea

E-mail: (H. L.) hyosunlee@knu.ac.kr; (S.-H. L.) slee24@dgu.ac.kr; (E. S.) eyseo@uc.ac.kr

KEYWORDS. supramolecular polymer templates; BTA-oligomers; molecular dispersity; self-assembly; nanoparticle synthesis

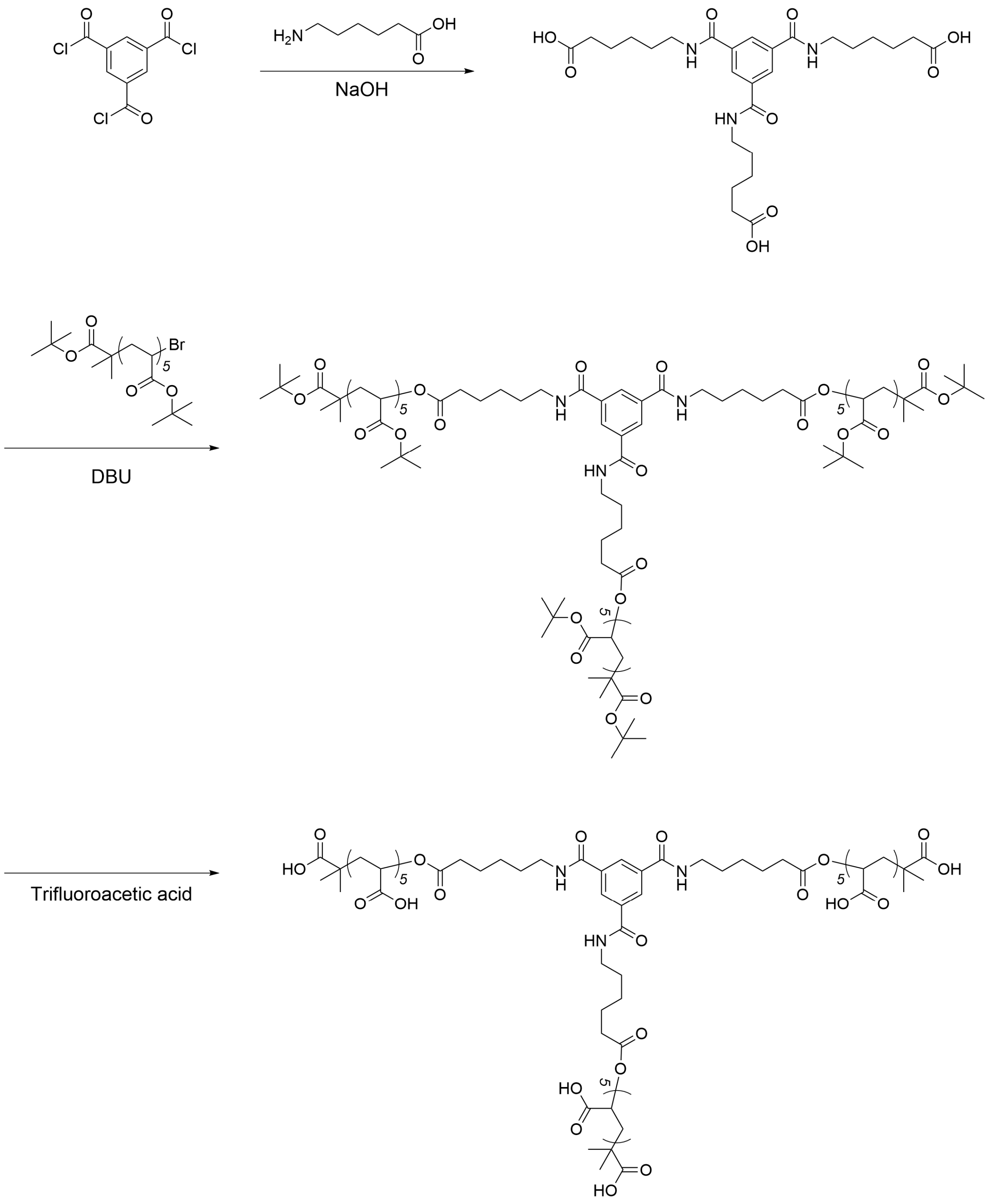

Scheme S1. Synthetic procedure for the three-armed BTA-oligo(AA)$_3$ molecule.

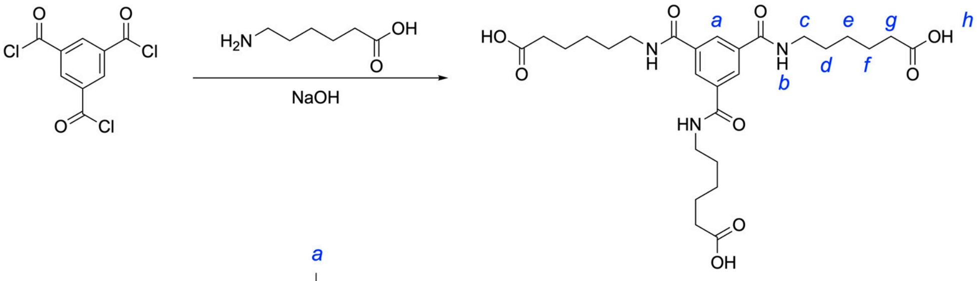

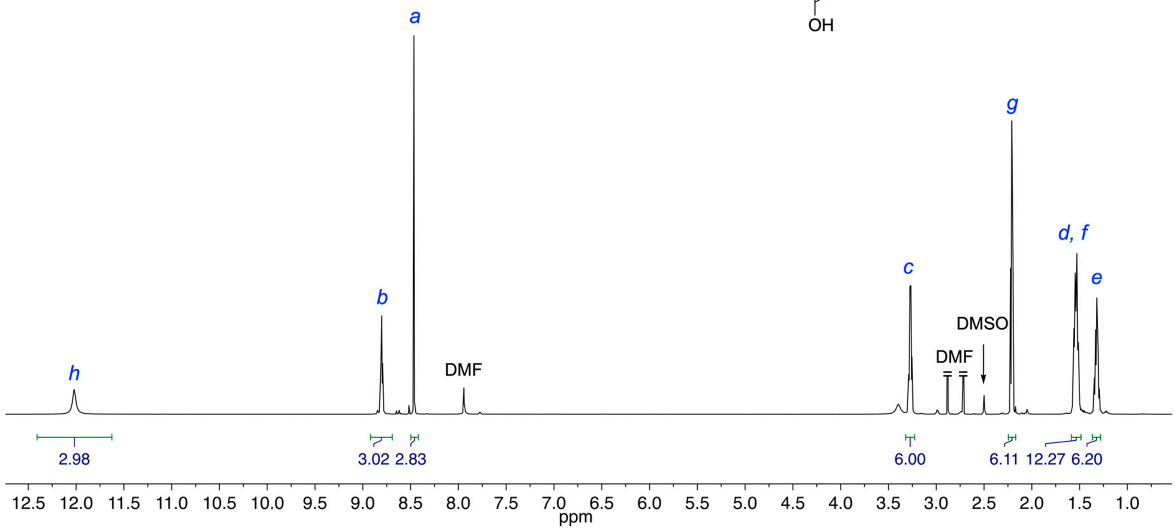

Figure S1. ^{1}H NMR spectrum of 6,6',6''-((benzene-1,3,5-tricarbonyl)tris(azanediyl))trihexanoic acid.

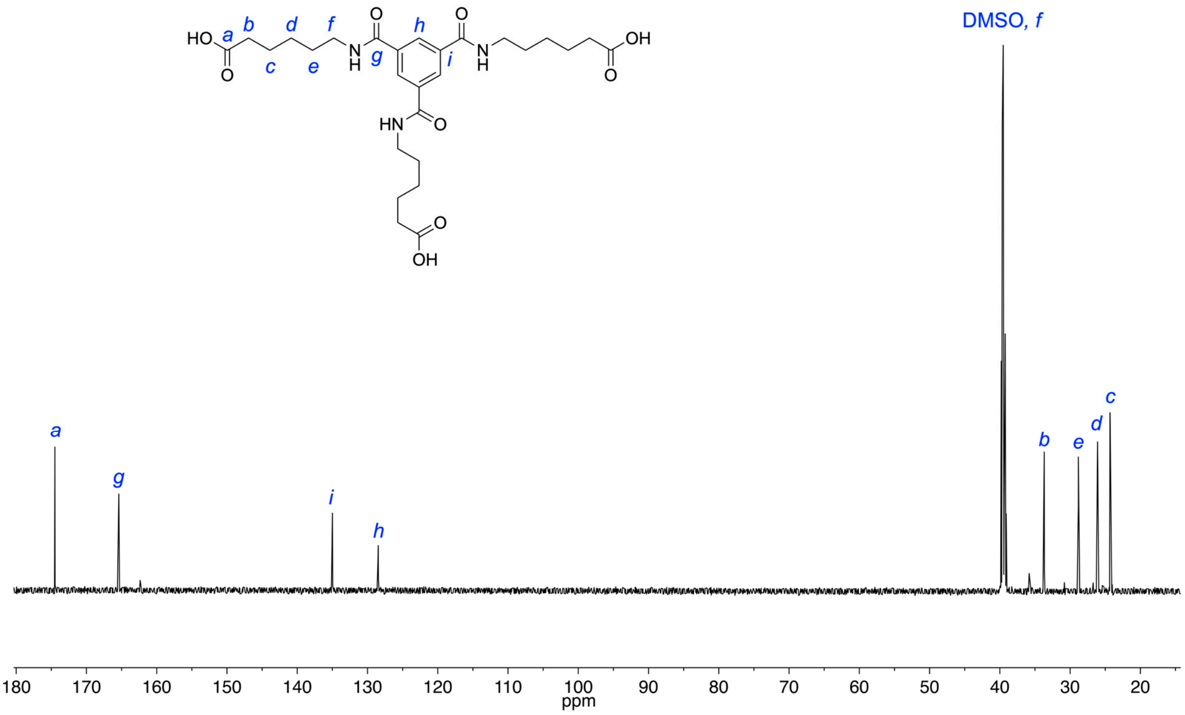

Figure S2. ^{13}C NMR spectrum of 6,6',6''-((benzene-1,3,5-tricarbonyl)tris(azanediyl))trihexanoic acid.

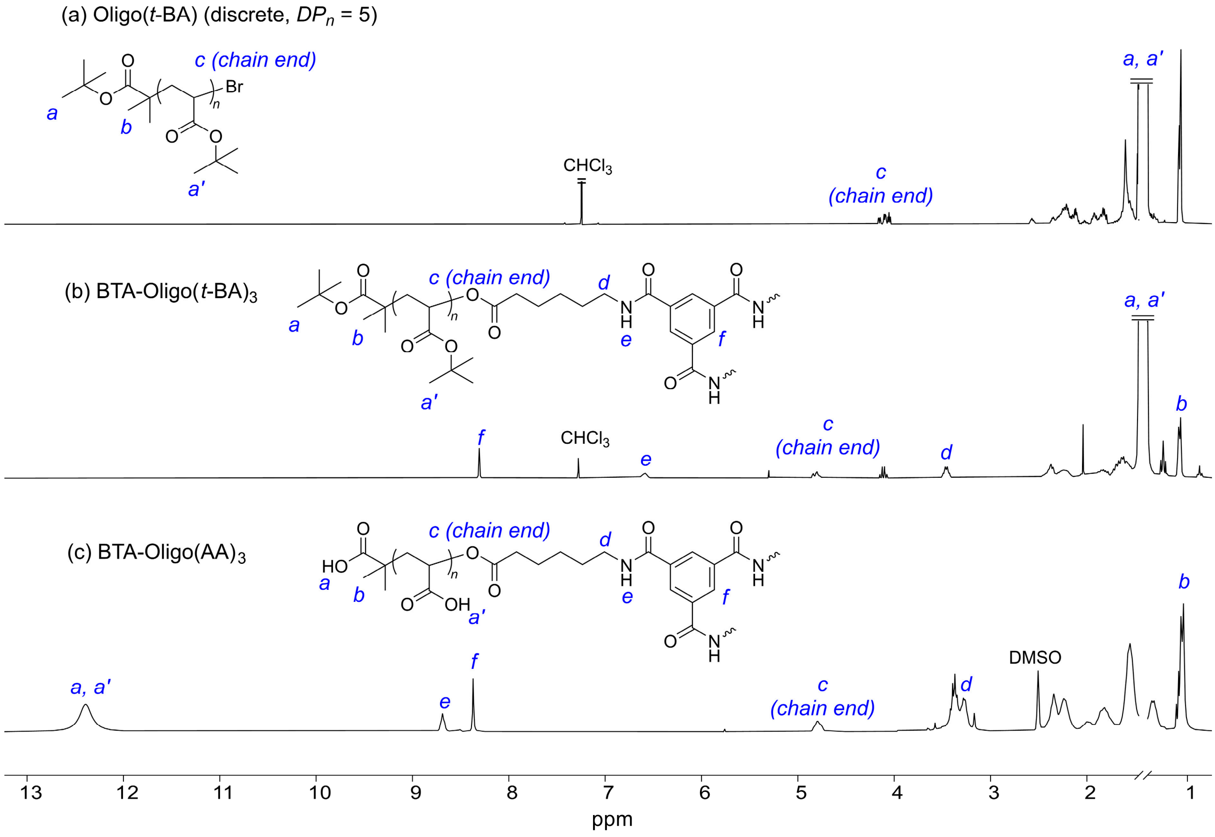

Figure S3. ^{1}H NMR spectra of (a) discrete oligo(*t*-BA) with $DP_n = 5$, and (b) discrete BTA-oligo(*t*-BA)$_3$ and (c) discrete BTA-oligo(AA)$_3$ synthesized using discrete oligo(*t*-BA) with $DP_n = 5$.

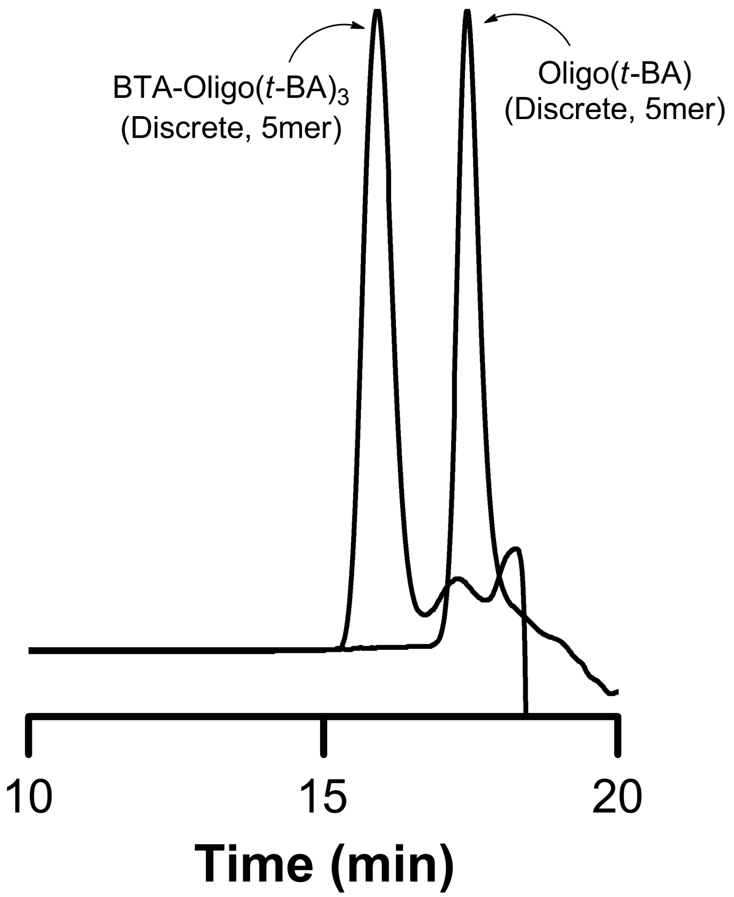

Figure S4. SEC curves for discrete oligo(*t*-BA) and its corresponding discrete BTA-oligo(*t*-BA)$_3$.

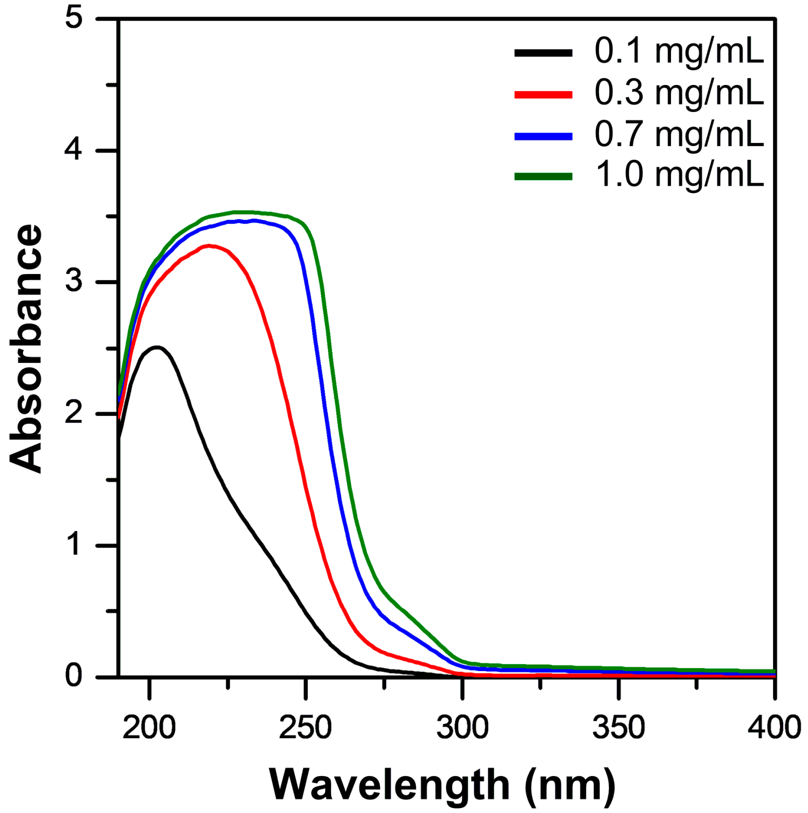

Figure S5. UV-vis absorption spectra of discrete BTA-oligo(AA)$_3$ at different concentrations (0.1, 0.3, 0.7, and 1.0 mg/mL), showing the effect of concentration on optical behavior.

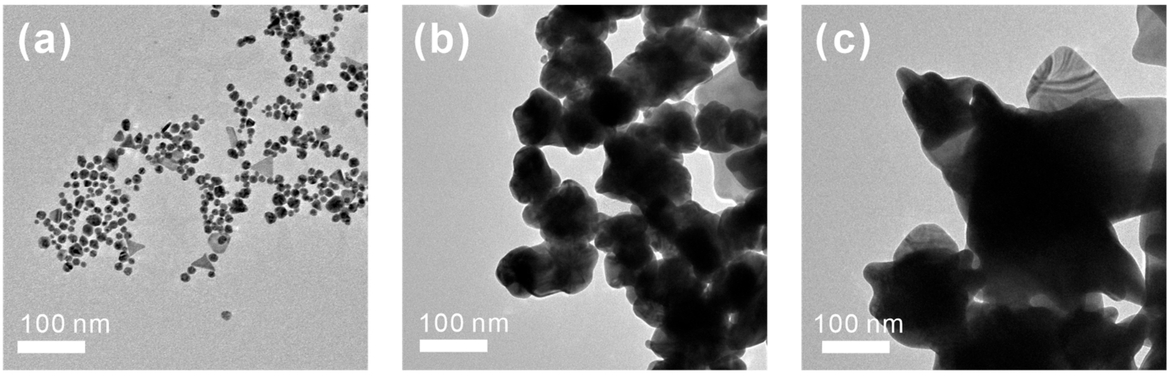

Figure S6. TEM images showing structural variations in gold nanomaterials synthesized using discrete BTA-oligo(AA)$_3$ templates. The images illustrate the effect of precursor and oligomer concentrations on nanostructure formation. The concentrations of discrete BTA-oligo(AA)$_3$ templates in aqueous solution are: (a) 0.1 mg/mL, (b) 1.0 mg/mL, and (c) 2.0 mg/mL.
